# Disease Activity and Psychosocial Factors Associated with Heath-Related Quality of Life in Patients with Crohn’s Disease: A Cross-Sectional Study

**DOI:** 10.3390/healthcare14040432

**Published:** 2026-02-09

**Authors:** YoonJi Roh, Hye-Ah Yeom

**Affiliations:** College of Nursing, The Catholic University of Korea, Seoul 06591, Republic of Korea; fjrioled@catholic.ac.kr

**Keywords:** Crohn’s disease, coping, health-related quality of life, nursing, post-traumatic growth, self-management, disease activity

## Abstract

**Background:** Crohn’s disease has a pattern of recurrent remissions and flare-ups which makes patients experience psychological complications; however, few studies have been conducted to identify intra-personal factors associated with health-related quality of life in individuals with Crohn’s disease. This study aimed to explore how disease activity, coping, and post-traumatic growth were associated with health-related quality of life in patients with Crohn’s disease. **Methods**: A cross-sectional study was conducted using self-reported questionnaires. Of the 227 adult patients recruited from a Crohn’s disease online support group in Korea, 219 were included in the final analysis. Measurements included the Harvey–Bradshaw Simple Index, the Korean version of the Short Inflammatory Bowel Disease Questionnaire, the Korean version of the Coping and Adaptation Processing Scale Short-Form, and the Korean version of the Post-traumatic Growth Inventory. Data were analyzed using descriptive statistics, independent *t*-tests, one-way ANOVA, Pearson’s correlation, and multiple linear regression. **Results**: The mean score of health-related quality of life was 4.10 out of 7 points, and the subdomain of emotional health showed the lowest score. Most participants were classified as having mild disease activity. The multiple regression analysis revealed that disease activity was significantly associated with health-related quality of life, which accounted for 31.2% of the total variance. Coping and PTG were not significantly associated with health-related quality of life. **Conclusions**: Disease activity was a significant factor associated with the health-related quality of life of Crohn’s disease patients. It is important to control the disease activity level in Crohn’s disease patients through self-management strategies. Maintaining a low stage of disease activity can be a crucial component of nursing care plans for enhancing health-related quality of life in individuals with Crohn’s disease.

## 1. Introduction

Crohn’s disease is one of the inflammatory bowel diseases (IBDs) that affects the entire gastrointestinal tract from the mouth to the anus [1,2]. The global incidence of IBDs is 4.45 per 100,000 people, and the incidence rate of Crohn’s disease has increased consistently during the past decade [3]. Although Crohn’s disease has been most common in Western countries, including North America, Europe, and Oceania, the incidence rates have recently stabilized in these regions, while rapidly increasing in countries in Latin America, East Asia, and the Middle East [4]. The prevalence of the disease tends to peak in adulthood, particularly between the 20s and early 30s [1,2]. In Korea, similarly to global trends, the proportion of medical visits for Crohn’s disease by age group is highest among those in their 20s, who account for 30.4% of visits, followed by those in their 30s at 22.6% [5].

Crohn’s disease is characterized by symptoms such as diarrhea, abdominal pain, anorexia, and low-grade fever [1,2]. The disease pattern of sudden remissions and active periods causes psychological distress in the patient [6]. In particular, Patients in their 20s and 30s, who are expected to have an active social life, experience drastic physical and psychosocial changes due to various clinical symptoms and complications that lead to psychological loneliness and isolation [7]. Compared with ulcerative colitis, another type of inflammatory bowel disease with symptoms similar to Crohn’s disease, patients with Crohn’s disease undergo frequent surgeries and report a lower quality of life than patients with ulcerative colitis [8].

Health-related quality of life (HRQoL) represents an individual’s personal evaluation of the physical and psychosocial aspects of their life that are affected by a disease or condition [9,10]. HRQoL reflects the patient’s perception of their condition and interventions to treat it, so it is a critical determinant in assessing the effectiveness of treatment methods and nursing interventions [9,11]. Previous studies have reported various correlates of the HRQoL of patients with Crohn’s disease, including pain and anxiety, Crohn’s disease activity, and depression symptom clusters [12,13,14]. Specifically, disease activity has been identified as a critical factor associated with the quality of life of patients with Crohn’s disease [13,15].

Disease activity, a significant predictor of HRQoL in this study, describes the type and severity of invasions in each organ of the body at the measurement time [16]. The physical, psychosocial, and socioeconomic problems faced by people with Crohn’s disease, from the onset of the illness to the end of treatment, are influenced by disease activity and can themselves exacerbate inflammatory symptoms, resulting in functional disability and negative emotions [17]. This cycle significantly impairs the quality of life of people with Crohn’s disease [18]. It is known that the more active the disease, the worse the HRQoL in Crohn’s disease [13,15]. In a study of patients with ulcerative colitis, those in remission showed better HRQoL than those in the active stages of inflammation [8,19]. Therefore, it is crucial to help patients reduce their disease activity to improve their quality of life.

Although Crohn’s disease is not fatal, it is a chronic illness that requires ongoing management throughout life. When it is not managed effectively, patients experience physical side effects and psychological complications, and often report difficulties with school or work [20]. Therefore, it is important to effectively cope with the physical and psychological crises that can occur during the recurrent course of the disease.

Coping refers to a range of adaptive responses to stress, and can involve ongoing cognitive and behavioral efforts to manage external or internal situations that exceed an individual’s capabilities [21]. Although active coping with disease is known to be associated with higher HRQoL in disease conditions such as breast cancer [22,23] and stroke [24,25], evidence specific to Crohn’s disease is still limited. Given the rapid physical and social changes that patients with Crohn’s disease can experience during an uncertain disease process, it is necessary to explore the potential association between coping and HRQoL in this population.

Post-traumatic growth (PTG) refers to a subjectively perceived positive psychological change by adapting to stressful situations and overcoming traumatic experience [26]. PTG is characterized by positive psychological changes that result from a traumatic event, and those positive changes and growth not being temporary but leading to real life changes [27]. A large number of Crohn’s disease patients require surgical treatment because of complications, with nearly half of patients undergoing surgery within 10 years of diagnosis [10]. The proportion of Crohn’s disease patients undergoing bowel resection has continuously increased from 8.96% in 2010 to 16.37% in 2018 [28], and the risk of surgery 10 years after diagnosis is as high as 46.6% at 10 years and 33.3% at 5 years [29]. Patients with Crohn’s disease who undergo surgery and reoperation often report negative body image perceptions due to surgical scars and the use of ostomy for defecation [30]. Therefore, surgery is still a traumatic experience for patients with Crohn’s disease. On the other hand, traumatic experiences such as surgery can also lead to personal growth. Patients with Crohn’s disease who undergo such experiences might initially feel fear, helplessness, and pain, but they can emerge from their ordeals with renewed resilience and a willingness to face and embrace life positively [31]. Studies have shown that high levels of PTG were associated with high quality of life among cancer patients [32,33]. Although Crohn’s disease and cancer represent different pathologies, the shared characteristics of both diseases, such as experiencing frequent surgeries and chronic nature of the disease progress, suggest that PTG may be relevant to the HRQoL of patients with Crohn’s disease. However, this association for Crohn’s disease is not yet well understood, and research on the relationships among disease activity, coping strategies, PTG, and HRQoL in patients with Crohn’s disease is still limited. Therefore, this study aims to explore the associations of disease activity and exploratory psychosocial factors, including coping and PTG, with HRQoL among patients with Crohn’s disease.

## 2. Materials and Methods

### 2.1. Study Design and Participants

This was a cross-sectional descriptive study. The participants of this study were recruited from the Korean online patient support community using a convenience sampling method. The online recruitment method was chosen to ensure feasibility and to facilitate access to dispersed patient population. The number of subjects was calculated based on the G power 3.1 program with multiple regression [34]. With minimum effect size (f^2^ = 0.15), a significance level of 0.05, a statistical power of 0.80, and 7 predictors included in the regression model, the minimum acceptable sample size was 153. In this study, a total of 227 participants were initially recruited. After excluding incomplete survey questionnaires, the final sample contained 219 participants, which satisfied the minimum required sample size. The inclusion criteria were (a) age ≥ 18 years old; (b) diagnosed with Crohn’s disease; (c) receiving outpatient treatment; and (d) volunteering to participate in the study. The exclusion criteria were (a) patients who had been diagnosed with colorectal cancer; (b) patients who have concurrent gastrointestinal disorders (e.g., ulcerative colitis, indeterminate colitis, celiac disease, or Behcet’s enteritis); and (c) patients with a language disorder or communication problem.

Data were collected from December 2022 to January 2023 via an online patient support community, the Crohn’s Disease Patient Association, in which a recruitment notice was posted. Although the sample represented community-dwelling and digitally engaged outpatients, they did not fully reflect the Crohn’s disease population. An online survey with Google Forms was conducted. The announcement flyer contained the purpose of the study, eligibility criteria for participants, data collection period and procedures, rewards for study participation, and a link to the survey site. Participation was voluntary and anonymous. Participants were asked to complete the survey if they agreed to participate, and were instructed to exit the survey if they did not. The questionnaire took 15 to 20 min to complete. To prevent duplicate responses, submissions were restricted to one response per participant. In addition, to prevent incomplete responses, all items were set as mandatory, as the participants could not proceed to next item without providing every response.

### 2.2. Measurements

#### 2.2.1. Demographic and Clinical Characteristics

Demographic characteristics included gender, age, marital status, level of education, religion, monthly income, and occupation. Clinical characteristics included disease activity, disease duration (e.g., how long patients had been diagnosed with Crohn’s disease), experience of hospitalization, number of hospitalizations, experience of surgery, number of surgeries, and presence of ostomy.

#### 2.2.2. Disease Activity

Disease activity in Crohn’s disease was measured using the Harvey–Bradshaw Simple Index (HBI), which was developed to measure the level of disease activity through the patient’s answer of five questions [35,36]. As this study was conducted via an online survey, the self-reported version of the HBI was used. The scale does not include any biochemical tests [36] and has been used in clinical setting due to its simplicity of calculation [37]. The HBI consists of five items that evaluate the respondent’s subjective perception on general well-being (scored from 0 to 4), the severity of abdominal pain (scored from 0 to 3), the number of liquid stools per day (scored 1 point per episode), abdominal masses (scored from 0 to 3), and the presence of complications (scored 1 point per each). Regarding accessing abdominal mass, participants were instructed to report based on self-palpation or recent medical consultation. Previous studies have showed a high correlation between the self-reported HBI and physician-reported scores [38,39]. The total score was calculated by adding the points for each question, and disease activity was categorized into four stages: remission (<5), mild activity (5 to <8), moderate activity (8 to <16), and severe activity (≥16) [40].

#### 2.2.3. Coping

The Korean version of the Coping and Adaptation Processing Scale-Short Form (KCAPS-SF) was used to measure coping strategies [40]. It was adapted from a shortened version of CAPS [41], based on Roy’s adaptation model, and consists of 15 items. Each item is measured on a 4-point Likert-type scale and the total score ranges from 15 to 60. The score is calculated by adding the points for each question and reverse-scoring three negative items. The higher the total score, the greater degree of adaptation to the environment [40]. Cronbach’s alpha of the scale was 0.72 in this study.

#### 2.2.4. Post-Traumatic Growth

The Korean version of the Post-traumatic Growth Inventory (K-PTGI) was used to measure post-traumatic growth [26,42] in this study. The K-PTGI consists of a total of 16 items with four subdomains: Personal strength (6 items), relating to others (5 items), new possibilities (3 items), and spiritual change (2 items). Each item is scored on a 6-point Likert scale, with 0 being “not at all” and 5 being “a lot”, indicating the extent to which the individual has experienced positive change since their traumatic experience. In this study, the traumatic experience was defined as the diagnosis of Crohn’s disease or experience of Crohn’s disease-related surgery. The score is calculated by adding the points for each question and the total score ranges from 0 to 90. A high total score indicates a large number of positive changes experienced. Cronbach’s alpha of the scale was 0.92 in this study.

#### 2.2.5. Health-Related Quality of Life

Health-related quality of life was measured using the Korean version of the Short Inflammatory Bowel Disease Questionnaire (K-SIBDQ) [43] https://research.mcmaster.ca/industry-investors/technologies-available-for-licensing/request-for-license/ (accessed on 17 November 2025) The Short Inflammatory Bowel Disease Questionnaire (SIBDQ) has been widely used to assess HRQoL among patients with IBD, including Crohn’s disease, in clinical practice and research [44,45], and the validity of the scale has been reported [46]. The scale assesses HRQoL during the preceding 2 weeks. It consists of 10 questions with four subdomains: bowel symptoms, systemic symptoms, emotional health, and social function. Each item is answered on a 7-point scale ranging from 1, “I have very serious problems,” to 7, “I have no problems at all,” with higher scores indicating better quality of life. The final score is calculated by adding the points and dividing the result by the number of questions, for a possible range of 1 to 7. Cronbach’s alpha of the scale was 0.92 in this study.

### 2.3. Ethical Considerations

Ethical approval was obtained from the Institutional Review Board (IRB) of the Catholic University of Korea (MC22QASI0088). Subjects were informed that they could refuse to participate or withdraw their participation at any time during the study. The survey questionnaire included a statement about subject anonymity and affirmed that the contents of the survey would be used solely for research. Written informed consent was exempted because the survey was completely anonymous and conducted via online. The participants’ contact information would be used to provide the promised rewards. An online gift voucher was provided to the participants.

### 2.4. Statistical Analysis

The data were analyzed using IBM SPSS program for Windows, Version 27.0.(Armonk, NY, USA). Descriptive statistics were used to present demographic and clinical characteristics, coping, post-traumatic growth, and HRQoL in patients with Crohn’s disease. Statistical assumptions were examined prior to data analysis. Normality was assessed using skewness and kurtosis. Regarding the “number of hospitalizations” variable, which showed a skewed distribution, two approaches were adopted to confirm statistical validity. For group comparisons (*t*-test or ANOVA), the variable was categorized to mitigate the impact of its skewed distribution, and parametric analyses were considered appropriate based on the central limit theorem, given the sufficiently large sample size (N = 219). Linearity was confirmed through scatter plots. For the multiple regression analysis, the homoscedasticity and independence of residuals were assessed using residual scatter plots and the Durbin–Watson statistic, respectively.

Differences in HRQoL by demographic and clinical characteristics were analyzed by independent sample *t*-tests or one-way analysis of variance (ANOVA). As significant differences were found in ANOVA, the Scheffé post hoc test was conducted to identify group differences. The correlations among disease activity, coping, PTG, and HRQoL were examined by Pearson’s correlation. Finally, multiple linear regression analysis was used to identify the factors associated with HRQoL. Regression variables were selected based on significance (*p* < 0.05) in univariate analyses. Multicollinearity among the independent variables was examined using the Variance Inflation Factor (VIF). All VIF values (ranged from 1.071 to 1.851) were below the cutoff value of 10, indicating no significant multicollinearity issues. A *p*-value of <0.05 was considered statistically significant.

## 3. Results

### 3.1. Demographic and Clinical Characteristics

Among the 219 Crohn’s disease patients, 51.1% were women and 48.9% were men. The mean age was 37.68 ± 9.78 years, and 44.3% of the patients were between 30–39 years old. About half of the participants (54.3%) reported not having a spouse or partner, and 76.3% were undergraduates or of higher education level. Of the respondents, 68.5% denied a religious affiliation, and 43.4% were salaried workers. An average household income between USD 2000 and USD 2700 per month was reported by 38.4% of the participants. Over half of the patients (72.2%) were in the remission or mild disease activity stage, with 27.8% reporting moderate or severe disease activity and one patient reporting a severe activity score of 16 or more. The mean duration of Crohn’s disease was 3.36 ± 3.27 years, with 60.3% having been diagnosed for less than 3 years. More than half (54.3%) of the patients had been hospitalized, and the mean number of hospitalizations was 2.16 ± 13.75. Approximately one out of five patients (24.2%) reported that they had undergone surgery, with a mean number of surgeries of 0.37 ± 0.93. A total of 64.8% had never had an ostomy (Table 1).

### 3.2. Levels of Coping, Post-Traumatic Growth, and Health-Related Quality of Life

The levels of coping, PTG, disease activity, and HRQoL are shown in Table 2. The mean total score of coping was 2.77 ± 0.34 out of 4. The subdomain with the lowest mean score was alert processing (2.56 ± 0.44), followed by physical and fixed (2.74 ± 0.62), positive and knowing-based coping (2.78 ± 0.45), and resourceful and focused coping (2.90 ± 0.45). The mean score of PTG was 48.79 ± 12.40 out of 80. The mean total score for HRQoL was 4.10 ± 0.78 out of 7. The subdomains with the lowest level of HRQoL was emotional health (3.88 ± 0.87), followed by systemic symptoms (4.15 ± 0.94), bowel systems (4.20 ± 0.86), and social function (4.22 ± 1.08).

### 3.3. Correlation Among HRQoL, Coping, PTG, and Disease Activity

HRQoL was negatively correlated with disease activity (r = −0.55, *p* < 0.001), and PTG was positively correlated with coping (r = 0.66, *p* < 0.001) (Table 3).

### 3.4. Demographic and Clinical Characteristics with Differences in HRQoL

Significant differences in HRQoL were found regarding monthly income (*p* = 0.023), religion (*p* = 0.035), disease activity (*p* < 0.001), experience of hospitalization (*p* = 0.015), and the number of hospitalizations (*p* < 0.001). Regarding the number of hospitalizations, those who had experienced three or more hospitalizations had a significantly lower HRQoL than those who had no hospitalization experience or one to two hospitalizations (*p* < 0.001). Additionally, patients with higher disease activity showed significantly lower HRQoL (Table 1).

### 3.5. Multiple Linear Regression of the Factors of HRQoL

The multiple linear regression analysis included variables that significantly differed or correlated in univariate analyses, as shown in Table 4. The significant variable associated with HRQoL of Crohn’s disease patients was disease activity. Disease activity was negatively associated with HRQoL (β = −0.537, *p* < 0.001), and the model accounted for 31.2% of the total variance.

## 4. Discussion

This study was conducted to explore how disease activity, coping, and post-traumatic growth influence HRQoL in patients with Crohn’s disease. In this study, the mean HRQoL was 4.10 out of 7, indicating that patients with Crohn’s disease were moderately satisfied with their quality of life. By subdomain, the HRQoL level was the lowest in the domain of emotional health, which can be attributed to the chronic nature of inflammatory bowel disease characterized by recurrent periods of remission and activity. It can cause psychological stress in the form of constant anxiety about worsening symptoms [47]. Anxiety and stress can trigger the release of neurotransmitters such as serotonin that can spark an inflammatory response and exacerbate Crohn’s disease [48]. Thus, it is essential to consider the emotional distress experienced by patients who are managing Crohn’s disease and implement interventions to alleviate it and thus improve their quality of life.

In this study, disease activity was found to affect the HRQoL in patients with Crohn’s disease, which is consistent with the findings of previous studies [13,15] that found that the HRQoL of Crohn’s patients decreased as disease activity increased. Therefore, it is essential to maintain disease activity at an appropriate level to improve HRQoL in Crohn’s disease patients. In this study, most participants (72.1%) were categorized as being in remission or mild activity, indicating that the severity of Crohn’s disease in this group of outpatients was generally low. Previous research has shown that an increase in physical symptoms is associated with greater limitation of social activities by patients with Crohn’s disease, which can lead to psychological distress such as depression [7,17]. Those negative emotions can themselves have a detrimental effect on disease progression and decrease HRQoL [12,13,14]. Therefore, to improve the HRQoL of patients with Crohn’s disease, it is important to help patients manage their symptoms experienced during active or remission periods. Furthermore, it is necessary to identify factors that mediate the relationship between disease activity and HRQoL and further specify the mechanisms by which disease activity reduces HRQoL.

In this study, the mean score for coping was 2.77 out of 4, indicating a moderate level of coping in patients with Crohn’s disease. In general, more effective coping skills correlate with better psychological adjustment and higher quality of life in patients with chronic illnesses such as chronic kidney disease [49] and cancer [50]. However, in this study, the relationship between coping and HRQoL in patients with Crohn’s disease was not significant. It is difficult to generalize the results of this study because few studies have been conducted on coping in patients with Crohn’s disease. Considering that the subjects of this study were participants in a support group for Crohn’s disease patients, they are likely to have been engaging in positive social interactions and sharing experiences with other patients, rather than coping on an individual level. Participation in online support groups is known to help patients experience an increase in empowerment and self-esteem and a decrease in stress and social isolation [51]. As social support empowers individuals to manage and reduce stress, which positively affects the quality of life of people with Crohn’s disease [14], it is necessary to further explore the effects of coping on HRQoL in Crohn’s disease outpatients from the perspective of their social context rather than individual coping skills. Considering the chronicity of Crohn’s disease, it is also necessary to develop a disease-specific coping tool tailored to the remission and flare-up cycle characteristic of Crohn’s disease.

PTG is a phenomenon that occurs as a cognitive process of perceiving a traumatic experience and trying to find its meaning [31]. In this study, the average level of PTG was moderate, with a mean score of 48.79. The results of the study showed that PTG was not a significant factor affecting HRQoL in Crohn’s disease patients, which is inconsistent with our assumption. This finding might be attributable to several factors, such as the relatively young age of the patients, the low rate of surgery, or the level of resilience. The average age of the participants was 38 years, and 68% of the patients were currently employed. This young working group characteristic might contribute to their perception that Crohn’s disease treatment has a relatively minor effect on their daily lives, which could differ from the perceptions of older patient groups. As only 24.2% of the patients reported having undergone surgery in this study, most participants may have not been exposed to traumatic surgical events, which would limit the influence of PTG on their quality of life. Considering that other psychological and social factors may be confounded in the relationship between PTG and HRQoL, it is recommended that the influence of PTG on HRQoL be further examined in future studies of Crohn’s disease patients with diverse ages and disease severity.

While this study focused on the direct associations between psychological factors and HRQoL, coping and PTG may be distal or context-dependent factors. For instance, coping and PTG could influence HRQoL indirectly through mediating mechanisms by reducing emotional distress, enhancing self-efficacy, or facilitating adherence to treatment. Further longitudinal studies including psychological and clinical mediators are needed to clarify these pathways.

### 4.1. Limitations

Several limitations of this study should be acknowledged. First, patients who participate in online support groups are more likely to share disease-related information, adopt better disease management strategies, and receive social support. It potentially leads to higher HRQoL scores compared to those who do not participate in support groups, such as hospitalized patients. Furthermore, as data were collected from a community-dwelling outpatient Korean online support group, this result may not be completely generalized to a more acute population such as hospitalized patients or to patients of different ethnicities or cultures. Another limitation is the short study period (December 2022 to January 2023). Because Crohn’s disease is characterized by recurrent cycles of remission and flare-ups that may be associated with multiple clinical and behavioral factors (e.g., smoking, dietary patterns and anxiety), these findings may not fully represent the variability of disease activity. Additionally, disease activity was assessed relying only on the self-reported HBI without any objective markers. While the self-reported HBI is a validated tool and demonstrated high correlation with physician-assessed HBI scores, the lack of clinical confirmation (e.g., biomarker data) may have introduced subjective bias and could have limited the accuracy of disease activity assessment. Furthermore, several potential confounders were not assessed in this study, including psychological comorbidities (e.g., depression or anxiety) and treatment-related factors such as the type and intensity. In particular, participants’ medication use was not accounted for, which is a critical factor in managing Crohn’s disease and should be evaluated in further research. Lastly, due to the cross-sectional study design, causality among variables cannot be established.

### 4.2. Implications for Practice

Despite the limitations, this study contributes to the existing body of literature by extending the knowledge about patients with Crohn’s disease. Implications of the study include both practices for health professionals and research. In this study, disease activity was the major determinant factor affecting HRQoL in Crohn’s disease, indicating that managing disease activity is important to improve the HRQoL of patients with Crohn’s disease. To engage in effective self-management, health professionals, including nurses, should educate and empower the patients with Crohn’s disease to adhere to medical treatment. Adherence to medication and disease-specific medical therapy is also a critical component of Crohn’s disease management, as non-adherence to treatments may exacerbate disease activity and subsequently reduce HRQoL. Health professionals should implement personalized education of medical treatments based on each disease activity level and support patients to understand the importance of adherence to treatments. In future studies, structured education programs should be developed to help patients understand the characteristics of Crohn’s disease, leading to better disease activity control and enhancing HRQoL.

## 5. Conclusions

Based on the results, the level of HRQoL among community-dwelling patients with Crohn’s disease was moderate, and the lowest scores were reported in the subdomain of emotional health. Disease activity was significantly associated with HRQoL in this population. Therefore, maintaining a low stage of disease activity may contribute to better HRQoL in Crohn’s patients. Further research should identify detailed disease-specific coping processes to clarify the experiences of patients with Crohn’s disease.

## Figures and Tables

**Table 1:** Differences in HRQoL by demographic and clinical characteristics (N = 219).

Variables	Categories	Total		HRQoL	
		n (%)	M ± SD	M ± SD	t or F (*p*)
Gender	Women	112 (51.1)		4.19 ± 0.80	1.80 (0.073)
	Men	107 (48.9)		4.01 ± 0.75	
Age (years)	<30	45 (20.5)	37.68 ± 9.78	4.05 ± 0.81	0.69 (0.562)
	30–39	97 (44.3)		4.08 ± 0.83	
	40–49	55 (25.1)		4.21 ± 0.69	
	≥50	22 (10.0)		3.97 ± 0.69	
Marital status	Single	116 (53.0)		4.12 ± 0.82	0.51 (0.600)
	Married	100 (45.7)		4.09 ± 0.73	
	divorced/widowed	3 (1.3)		3.67 ± 0.68	
Education	<Undergraduates	52 (23.7)		4.13 ± 0.66	0.40 (0.692)
	≥Undergraduates	167 (76.3)		4.09 ± 0.81	
Religion	Yes	69 (31.5)		3.91 ± 0.93	−2.14 (0.035)
	No	150 (68.5)		4.18 ± 0.68	
Occupation	Salaried worker	95 (43.4)		4.23 ± 0.84	1.45 (0.186)
	Housewife	43 (19.6)		4.04 ± 0.67	
	Self-employed	30 (13.7)		4.02 ± 0.68	
	Student	10 (4.6)		4.01 ± 0.72	
	Public Official	10 (4.6)		3.62 ± 0.77	
	Profession	10 (4.6)		4.11 ± 0.66	
	Farmer	4 (1.8)		4.52 ± 0.39	
	Others	17 (7.7)		3.86 ± 0.85	
Monthly income (USD)	<2000	71 (32.4)		3.94 ± 0.93	3.35 (0.023)
	2000–2700	84 (38.4)		4.27 ± 0.61	
	2700–3400	43 (19.6)		4.11 ± 0.78	
	≥3400	21 (9.6)		3.88 ± 0.78	
Crohn’s disease activity	Remission ^a^	74 (33.8)		4.61 ± 0.62	39.36 (< 0.001)
	Mild ^b^	84 (38.4)		4.00 ± 0.69	a > b > c ^†^
	Moderate/Severe ^c^	61 (27.8)		3.61 ± 0.70	
Disease duration (year)	<3	132 (60.3)	3.36 ± 3.27	4.06 ± 0.70	0.64 (0.536)
	3–5	41 (18.7)		4.06 ± 0.72	
	>5	46 (21.0)		4.24 ± 1.00	
Experience of hospitalization	Yes	119 (54.3)		3.98 ± 0.84	−2.46 (0.015)
	No	100 (45.7)		4.23 ± 0.68	
Number of hospitalizations	0 ^a^	100 (45.7)	2.16 ± 13.75	4.23 ± 0.68	15.61 (< 0.001)
	1–2 ^b^	89 (40.6)		4.18 ± 0.81	a > c ^†^, b > c ^†^
	≥3 ^c^	30 (13.7)		3.41 ± 0.65	
Experience of surgery	Yes	53 (24.2)		3.98 ± 0.95	−1.11 (0.269)
	No	166 (75.8)		4.14 ± 0.71	
Number of surgeries	0	166 (75.8)	0.37 ± 0.93	4.14 ± 0.71	1.78 (0.188)
	1	40 (18.3)		3.88 ± 0.79	
	≥2	13 (5.9)		4.27 ± 1.33	
Experience of ostomy	Yes	16 (7.3)		3.80 ± 0.67	1.76 (0.175)
	Restored	61(27.9)		4.04 ± 0.71	
	No	142(64.8)		4.16 ± 0.81	

Note. HRQoL, health-related quality of life. ^†^ Scheffé test: means with different lowercase letters are significantly different.

**Table 2:** Levels of disease activity, coping, and post-traumatic growth and health-related quality of life in patients with Crohn’s disease (N = 219).

Variables	Categories	M ± SD	Range
Coping	Total	2.77 ± 0.34	1~4
	Resourceful and focused	2.90 ± 0.45	1~5
	Positive and knowing-based	2.78 ± 0.45	1~4
	Physical and fixed	2.74 ± 0.62	1~3
	Alert processing	2.56 ± 0.44	1~3
Post-traumatic growth	Total	48.79 ± 12.40	0~80
	Personal strength	18.97 ± 5.27	0~30
	Relating to others	15.39 ± 4.50	0~25
	New possibilities	9.70 ± 2.49	0~15
	Spiritual change	4.73 ± 2.69	0~10
Health-related quality of life	Total	4.10 ± 0.78	1~7
	Social function	4.22 ± 1.08	1~7
	Bowel systems	4.20 ± 0.86	1~7
	Systemic symptoms	4.15 ± 0.94	1~7
	Emotional health	3.88 ± 0.87	1~7

**Table 3:** Correlations among health-related quality of life and the related variables (N = 219).

	Crohn’s Disease Activity	Coping	Post-Traumatic Growth	Health-Related Quality of Life
Crohn’s disease activity	1			
Coping	−0.08 (0.263)	1		
Post-traumatic growth	0.02 (0.732)	0.66 (<0.001)	1	
Health-related quality of life	−0.55 (<0.001)	0.05 (0.450)	−0.03 (0.712)	1

Note. Pearson’s correlation coefficient, *r*(*p*).

**Table 4:** Factors associated with health-related quality of life of patients with Crohn’s disease (N = 219).

Variable	B	S.E.	β	t	*p*	VIF
(Constant)	4.725	0.155		30.407	<0.001	
Religion (Yes = ref.)						
No	0.038	0.099	0.023	0.383	0.702	1.111
Monthly income USD (USD) (2700–3400 = ref.)						
<2000	−0.086	0.125	−0.052	−0.685	0.494	1.814
2000–2700	0.169	0.122	0.106	1.387	0.167	1.851
≥3400	0.010	0.174	0.004	0.057	0.954	1.382
Experience of hospitalization (Yes = ref.)						
No	0.106	0.091	0.068	1.157	0.248	1.094
Number of hospitalizations	0.005	0.003	0.086	1.476	0.141	1.071
Crohn’s disease activity	−0.126	0.014	−0.537	8.937	<0.001	1.145

Note. B, unstandardized coefficients; S.E., standard error; β, standardized coefficient; VIF, variance inflation factor. R^2^ = 0.334, Adjusted R^2^ = 0.312, Durbin–Watson = 1.646, F = 15.10, *p* < 0.001.

## Data Availability

The datasets used and analyzed during the current study are available from the corresponding author on reasonable request. The data are not publicly available due to privacy and ethical restrictions.

## Abbreviations

The following abbreviations are used in this manuscript:

IBD	Inflammatory bowel disease
HRQoL	Health-related quality of life
PTG	Post-traumatic growth
HBI	Harvey–Bradshaw Simple Index
KCAPS-SF	The Korean version of the Coping and Adaptation Processing Scale-Short Form
K-PTGI	The Korean version of the Post-traumatic Growth Inventory
K-SIBDQ	The Korean version of the Short Inflammatory Bowel Disease Questionnaire
SIBDQ	Short Inflammatory Bowel Disease Questionnaire
IRB	Institutional Review Board
ANOVA	One-way analysis of variance
